# Demographic Predictors of Complete Well-Being

**DOI:** 10.1186/s12889-022-13769-7

**Published:** 2022-09-06

**Authors:** Matthew T. Lee, Eileen McNeely, Dorota Weziak-Bialowolska, Karen A. Ryan, Kay D. Mooney, Richard G. Cowden, Tyler J. VanderWeele

**Affiliations:** 1 Human Flourishing Program, Harvard University, 12 Arrow St., Ste 100, Cambridge, MA 02138 USA; 2 Institute for Studies of Religion, Baylor University, Waco, TX 76798 USA; 3 Department of Environmental Health, T.H. Chan School of Public Health, Harvard University, Cambridge, MA USA; 4 AVANGRID, 180 Marsh Hill Road, Orange, CT 06577 USA; 5 Independent Researcher, Hartford, CT USA; 6 Departments of Epidemiology and Biostatistics, T.H. Chan School of Public Health, Harvard University, Cambridge, MB USA

**Keywords:** Well-being, Flourishing, Demographics, Race/ethnicity, Age

## Abstract

This paper examines demographic differences in flourishing, defined as “complete well-being” and consisting of six domains: emotional health, physical health, purpose, character strengths, social connectedness, and financial security. Results are based on a random, cross-sectional sample of 2363 survey respondents drawn from employees of a large, national, self-insured employer based in the United States. We found that well-being across domains tends to increase with age, although there are some variations. Results are similar across most domains for men and women, although women score higher on character strengths, while men had higher scores on financial security. Racial and ethnic differences were striking. Black employees score higher than the reference group (White employees) on the emotional, purpose, and character strengths domains, but considerably lower on financial security. Hispanics also score lower on financial security (though not as low as Blacks), but higher than Whites on purpose, character strengths, and social connectedness. Asians reported higher well-being than Whites across all domains except purpose.

**Supplementary Information:**

The online version contains supplementary material available at 10.1186/s12889-022-13769-7.

## Introduction

Human flourishing, or “complete well-being” [1], has been a long-standing concern in the humanities, most notably in philosophy, theology, and literature. Social scientific research has often focused on a more restricted subset of flourishing, such as “psychological well-being,” “subjective well-being,” “quality of life,” “life satisfaction,” or “happiness” [2–4]. The published works on these aspects of flourishing are voluminous, with one recent review of subjective well-being alone identifying over 140,000 articles [5]. Despite this high level of scholarly attention to related concepts, we actually know relatively little about the demographic predictors of different aspects of well-being.

Broadly conceived, the approach to well-being or flourishing taken here is “a state in which all aspects of a person’s life are good” and this is operationalized by five core domains which are “nearly universally desired” [1, 6] and are widely considered as ends in themselves: emotional health, physical health, meaning and purpose, character strengths, and social connectedness. A sixth domain -- financial security -- may be necessary to sustain the other domains over time. Recent research has established good psychometric properties for both a Flourish Index (FI), a measure obtained by summing the scores on items from each of the five core domains, and a Secure Flourish Index (SFI), which also includes the financial security domain [7, 8]. Research on a more detailed set of measures of secure flourishing based on 40 items (an expansion of the 12-item measure developed by VanderWeele [1]) demonstrated that all six domains are indeed highly valued and that valuing them is associated with actual self-reported well-being scores [6]. Building on this conceptual and empirical groundwork, the current paper investigates the demographic predictors of flourishing and secure flourishing using survey data from randomly sampled employees at a large healthcare services organization based in the United States. Previous research has generally not measured complete well-being, opting instead to address one or a handful of domains. We focus on the foundational demographic attributes of race/ethnicity, biological sex, and age that for most people are not altered by intentional choices over the life course, thus excluding such commonly used demographics such as, for example, marital status and educational attainment. By restricting analyses to ascribed rather than achieved statuses, we offer one of the first studies of how the “givens” of life shape complete well-being. We also avoid questions about causal ordering in cross-sectional studies by excluding predictors that might be affected by our outcomes. Our findings might be used to identify flourishing domains that could be targeted for enhancement in distinct sub-populations. Our results could inform future research on the relationships among ascribed and achieved statuses as well as flourishing.

### Demographics and Complete Well-Being

The demographic correlates of specific domains of well-being vary by domain [9] and across studies [10]. This complicates the predictions that we might make about complete well-being in our current sample. Even the relationship between positive and negative affect, two of the most basic building blocks of a single aspect of well-being (happiness), has been found to be more complicated than we might first assume [11]. It is not unreasonable to expect that well-being would involve a high level of positive affect and a low level of negative affect (e.g., a person who feels happy without also feeling anxious). But in fact, some people experience high levels of both at the same time, some cultural traditions normalize negative affect while others do not, and some aspects of well-being (e.g., social connections, purpose) may generate bad feelings rather than good ones [12]. As any parent who has spent months changing diapers in the middle of the night knows, a meaningful life may not always be a happy one [13]. Similarly, social connections may not foster well-being if personal relationships are strained rather than supportive, a finding that seems especially relevant for Black men who have experienced childhood adversity [14].

Some previous research has uncovered stability in the relationships between demographics and aspects of well-being. For example, one classic review that included data collected throughout the world found virtually no age, gender, or racial/ethnic differences in overall happiness, or psychological well-being [12]. However, other studies have found significant differences. Using data from the nationally representative Midlife in the United States (MIDUS) study, [15] found that Blacks had higher rates of “flourishing” (measured by “positive mental health”) compared to Whites on 12 out of 13 measures, including social growth, positive affect, life satisfaction, purpose, and social contribution. In that study, Whites had an advantage in only social acceptance. A review by Diener et al. [16] revealed a positive relation between “life satisfaction” and age, but a negative trend in positive affect, and argued that the latter finding may be a function of a decline in the intensity of feelings overall. Furthermore, they point out that older adults are better able to accommodate adversity and that the gap between expectations and reality may decline with age, leading to more contentment. More recently, Gutierrez et al. [17] (but see also Jebb et al. [18]; and Batz-Barbarich et al. [10]) found significant sex and age differences with regard to aspects of subjective well-being, which they explained through their connections with Big Five personality traits. Horley and Lavery’s [19] research noted that the relationship between age and subjective well-being was “equivocal,” while Stone et al. [20] found a U-shaped relationship between both global and hedonic well-being (i.e., life satisfaction and affect) and age, such that well-being rebounded at age 50 after a lull in middle adulthood. Interestingly, the latter study found no gender differences in the broader categories of well-being, but it did uncover greater sadness in middle age followed by a decline in this negative emotion among older adults, as well as higher levels of sadness, stress, and worry among women.

Other measures of well-being have been employed, including physical health, financial security, purpose, and social connections. Multivariate models combining physical and emotional health into “nearly complete health” or “complete health” (not to be confused with complete well-being) have revealed that this form of well-being is most prevalent among older adults (55–74 years), as well as among Blacks, men, the college educated, and those with higher household incomes [21]. Some aspects of psychological well-being tend to decline with age (e.g., purpose, personal growth), while others increase (positive relations, autonomy, mastery), and one (self-acceptance) did not vary over the life course [22]. Using a variety of community and national samples, researchers have found that women rather than men tend to have more positive relations with others and occasionally higher levels of personal growth. Blacks report lower levels of quality of life as well as financial security compared with Whites, and also higher levels of worrying, although they do report higher levels of self-efficacy [9].

The relationship between race and well-being outcomes is frequently mediated by social class (or other variables), perhaps requiring an “interactive model of race and class” [9]. A prominent line of research also argues the “declining significance of race” [23, 24] in the contemporary era. In this view, overt racial discrimination and inequalities have given way to class-based disadvantages such that lower financial well-being scores for Blacks, for example, are a function of a spatial mismatch between residence and good jobs, as well as a skills mismatch. Racial variations in well-being may now be primarily a function of class effects. In other words, racism has been at least partly replaced by economic dislocation [25]. Empirical support of this thesis has been extended to other ethnic groups in the U.S., including Asian and American Indian men but not Hispanics [26], although there is also support for the “persistent significance of race” [25]. Regardless of how this debate is resolved, it is abundantly clear that race and ethnicity are likely to be important predictors of domains of complete well-being, especially financial security. Understanding the relationship between race/ethnicity and financial security is therefore essential for efforts to promote flourishing.

Much of the well-being research to date has focused on Black/White comparisons, but the demographic landscape is much more diverse than this dichotomy implies. For example, Asian and Hispanic groups have distinctive cultural backgrounds which shape both positive and negative life outcomes [8, 27]. An emerging body of research has drawn attention to well-being outcomes that are better than expected for Hispanics and Blacks, suggesting that “strong ties to the labor market and family,” the “importance of family values,” or a “strong support and kinship system” might mitigate the harmful effects of adverse social conditions [9, 27]. This raises the possibility of an inverse relationship between financial security and social connectedness. Regardless of the mechanism, the outcomes for disadvantaged groups are so much more positive than expected in light of social stressors that the word “paradox” is frequently used to describe Hispanic all-cause mortality rates, Black positive mental health, Asian and Hispanic resilience despite physical illness, and many other relationships between minority groups and well-being [15, 28, 29]. Race and ethnicity may serve as a proxy for cultural practices, as some of the paradoxical well-being findings might be linked to either “informal social practices organized around notions of harmony” or “adversarial confrontation,” depending on the shared history of a particular group [27].

Although generally addressed to specific aspects of well-being rather than flourishing as a whole, the accumulated body of research does lead us to expect that some racial/ethnic groups might fare better than Whites, that we might find a U-shaped relationship between flourishing and age, that Blacks and perhaps Hispanics might have a comparatively low financial security score but comparatively higher scores on other measures, and that women may exhibit higher levels of connectedness than men. It is less clear whether we might find gender differences with regard to emotional health because previous work has not uncovered a disparity, although women’s higher levels of sadness, stress, and worry may impact this domain. Our sample is limited to working adults at a single employer and therefore may not replicate findings from community or national surveys. On the other hand, it provides an opportunity to examine how the “givens” of life shape complete well-being for adults from the same work context, thus providing a fresh vantage point from which to explore the extent to which demographic patterns remain robust across samples [9, 12].

## Method

### Participants

Participants were randomly sampled employees of a large, national employer that is one the 100 largest public companies in the United States, present in the market of health insurance for more than 100 years. The company’s yearly revenue amounts to $60 B with profits of $3.5 B. The workforce consists mostly of white-collar employees. The initial invitation and reminders to participate were sent to 15,000 employees in June 2018 through the work email system, with 52 participants randomly selected subsequently to receive a cash prize ranging from $100 to $1000 as an incentive. There were *N* = 2370 respondents, but only 2363 answered the demographic questions necessary for the analysis. Our results are therefore based only on these 2363 respondents. They were mostly females [82.4% vs. 74.5% for the entire target population] with the mean age of 43.3 years in the sample vs. 45.6 in the target population. Participants were also predominantly White, rather well-educated, office employees, which was also consistent with the profile of employees in the target population [30]. The study was approved by the Committee on the Use of Human Subjects, which serves as the Institutional Review Board for Harvard University.

### Measures

#### Criterion variables

We measured the following six domains of well-being with a total of 40 items (based on the strategy pursued in Lee et al. [6]): emotional health (e.g., life satisfaction, happiness, ability to deal with difficult emotions), purpose (e.g., meaning, purpose, worthwhileness), social connectedness (e.g., relationship satisfaction, feel understood, connected to and respected by community), character strengths (e.g., promote good, treat others kindly, delay gratification), physical health (e.g., not ill, work to improve health, not in pain), and financial security (e.g., not worried about expenses, have sufficient savings, engage in financial planning). Scores ranged from 0 ‘not important at all’ to 10 ‘extremely important.’ Following Weziak-Bialowolska et al. [8], we averaged the first five domains (without financial security, 34 items total) to create our FI and we averaged all six domains to create our SFI (the higher the score, the greater the flourishing). We will refer to scores on these two summative indices as indicators of flourishing and secure flourishing, respectively.

#### Demographic predictors

Three demographic variables reflecting the “givens” of life were used. These were race/ethnicity (White [reference category], Asian, Black, and Hispanic; other response options were not sufficiently prevalent to be kept in the analysis), gender (male [reference] and female; one “undefined” respondent was excluded), and age in four groups (less than 31 years [reference], between 31 and 40 years, between 41 and 50 years, and 51 years or older).

#### Analytic Strategy

Statistical analyses were performed with SPSS Version 25. After examination of descriptive statistics (mean values for the total sample and mean values by gender and race), we used linear regression in our primary analysis to assess the associations between demographics and specific domains of well-being, as well as our composite measures of complete well-being (FI and SFI). In separate models for each criterion variable, we regressed the domains of well-being, the FI, and the SFI on sex, age, and race/ethnicity simultaneously.

As noted, we restricted our analyses to ascribed rather than achieved statuses in order to understand how the “givens” of life shape complete well-being and we avoid concerns about causal ordering in cross-sectional studies by excluding predictors that might be affected by our outcomes. Controlling for sociodemographic, lifestyle, and work-related factors assessed later in life could change the regression coefficients for immutable characteristics like race/ethnicity (and therefore the interpretation of results) because they might be on the pathway from basic demographics (gender, age, and race/ethnicity) to well-being. However, as a robustness check, we repeated the primary analysis after entering additional covariates (sociodemographics: marital status, education, having children below 18 years old, owning a house; lifestyle: religious service attendance; work characteristics: job satisfaction, work-family conflict, number of days working from home, job insecurity). These results are presented in Supplemental Table S2.

We conducted a complete case analysis rather than using imputation for missing data. There were 2370 respondents in our study, but only 2363 respondents provided complete demographic information (reported in Table 1). Information on the prevalence of missing data for each variable is presented in Supplemental Table S1.

**Table 1:** Descriptive Statistics

***Predictors***	%
Gender (Female)	82.4
Race/Ethnicity	
White (ref)	72.2
Black	12.9
Hispanic	7.3
Asian	5.3
American Indian or Alaskan Native	0.5
Native Hawaiian or other Pacific Islander	0.1
Other	2.3
Age (years)	
< 31 (ref)	13.5
31 to 40	28.7
41 to 50	28.8
51+	29.0

*N* = 2363

## Results

Table 1 shows that 82% of our sample were female, 72% were White, and that respondents were divided evenly across the age categories except for the 13% who were under 31. All of these percentages suggest that our sample is roughly representative of the organization’s total workforce (total data available on request from the corresponding author). Table 2 reveals that our respondents reported a moderately high level of combined well-being: 7.66 on a 10-point scale for the FI and 7.43 for the SFI. The highest reported scores for the individual domains were for purpose (7.92) and character strengths (7.90). Financial security was ranked lowest (6.27). Disaggregation revealed important differences by race and gender on the individual domains of well-being, but only a 0.46 mean difference (MD) between the highest and lowest scores on the FI (range = 7.50 to 7.96). The SFI displayed a broader range of scores (highest MD = 0.83), highlighting the importance of the financial security domain (highest MD = 3.4, between Asian males and Black males). Our primary focus is on our regression results and we are providing these mean scores as background information. We therefore do not report significance testing in Table 2, but these results are available on request from the corresponding author.

**Table 2:** Means for Well-Being Domains (Scored 0–10) by Race and Gender

	Total	Asian Female	Asian Male	Black Female	Black Male	Hispanic Female	Hispanic Male	White Female	White Male
Emotional Health (*n* = 2273)	7.48 (1.57)	7.94	7.70	7.64	7.22	7.58	7.62	7.40	7.57
Purpose (*n* = 2276)	7.92 (1.58)	8.07	7.96	8.18	7.41	8.11	8.14	7.86	7.80
Social Connectedness (*n* = 2260)	7.34 (1.75)	7.74	7.21	7.30	6.86	7.62	7.45	7.33	7.22
Character Strengths (*n* = 2276)	7.90 (1.22)	8.20	7.62	8.16	8.08	8.24	8.12	7.85	7.64
Physical Health (*n* = 2244)	7.69 (1.73)	8.20	8.00	7.64	7.38	7.65	7.33	7.65	7.82
Financial Security (*n* = 2277)	6.27 (2.61)	7.41	7.99	5.20	4.59	5.54	5.41	6.36	6.89
Flourish Index (*n* = 2083)	7.67 (1.26)	7.96	7.73	7.73	7.50	7.83	7.76	7.61	7.59
Secure Flourish Index (*n* = 2060)	7.43 (1.31)	7.88	7.87	7.30	7.05	7.46	7.32	7.40	7.47

*Note*. Standard deviation in parentheses

Asian females reported the highest level of well-being on three of six individual domains, as well as the highest levels of both flourishing and secure flourishing, although Asian males had virtually the same score on the latter. Black males reported the lowest level of well-being on four domains and the lowest levels of both flourishing and secure flourishing, although we must add the caveat that there were only 22 respondents in this group. Despite the low number of respondents, the mean difference between the SFI scores for Black males and Asian females was statistically significant (*t* = 2.55, *p* = .012). Asian males had the highest financial security score (again, note the low number of respondents), while Black females scored highest on purpose. Financial security had the lowest scores of any of the domains for all race-gender combinations except for Asian men. Interestingly, Black females and Asian men had identical scores on the FI, but Asian men score a half point higher on the SFI. For Asian men and Asian women, we find the opposite pattern: similar scores on the SFI but a higher score on the FI for Asian women. A related shift occurs for Whites, although the MDs are quite small.

Table 3 presents the regression results for all six domains of flourishing as well as combined well-being (FI and SFI). Well-being across domains tends to increase with age relative to the reference category (under 31 years old) and a U-shaped relationship was evident for emotional health starting with the 31 to 40-year-old group. In fact, examination of standardized coefficients (betas) reveal that the strongest and most consistent predictor was the oldest age group (51+), which was positively related to all domains except physical health, as well as both the FI and SFI. Results were similar across most domains for men and women, although women score higher on character strengths, while men had higher scores on financial security. Racial and ethnic differences were striking. Black employees score higher than the reference group (Whites) on the emotional, purpose, and character strengths domains, but considerably lower on financial security. Hispanics also score lower on financial security (though not as low as Blacks), but higher than Whites on purpose, character strengths, and social connectedness. Turning to our two combined measures of well-being, we see that Hispanics scored higher on flourishing, but not secure flourishing. Asians reported higher well-being than Whites across all domains except purpose and this group was the only significant predictor of improved physical health. Asians also have higher FI and SFI scores than Whites.

**Table 3:** Regression Results for Demographic Predictors of Well-Being Domains

Predictor	Criterion Variable							
	Emotional Health	Purpose	Social Connectedness	Character Strengths	Physical Health	Financial Security	Flourish Index	Secure Flourish Index
Female								
B [95% CI]	−.119 [−.289, .050]	.087 [−.083, .257]	.148 [−.042, .339]	.195 [.063, .327]**	−.073 [−.263, .117]	−.493 [−.768, −.219]***	.037 [−.105, .179]	−.058 [−.205, .089]
Beta	−.029	.021	.032	**.061**	−.016	**−.071**	.011	−.017
Asian								
B [95% CI]	.517 [.220, .813]**	.271 [−.022, .565]	.343 [.014, .672]*	.270 [.045, .495]*	.451 [.121, .780]**	1.279 [.805, 1.753]***	.332 [.083, .581]**	.528 [.268, .789]***
Beta	**.072**	.038	**.044**	**.050**	**.057**	**.108**	**.058**	**.088**
Black								
B [95% CI]	.255 [.057, .452]*	.298 [.099, .497]**	−.030 [−.251, .191]	.348 [.194, .503]***	−.066 [−.288, .155]	−1.052 [− 1.369, −.734]***	.128 [−.041, .296]	−.071 [−.246, .104]
Beta	**.054**	**.063**	−.006	**.094**	−.013	**−.134**	.033	−.018
Hispanic								
B [95% CI]	.229 [−.021, .480]	.308 [.059, .558]*	.319 [.035, .602]*	.430 [.237, .624]***	−.096 [−.375, .183]	−.738 [−1.139, −.338]***	.239 [.028, .450]*	.073 [−.146, .291]
Beta	.038	**.051**	**.047**	**.092**	−.014	**−.074**	**.049**	.014
Age 31 to 40								
B [95% CI]	.218 [.007, .429]*	.286 [.073, .498]**	.182 [−.054, .417]	.148 [−.016, .311]	−.080 [−.315, .154]	−.286 [−.625, .054]	.141 [−.034, .317]	.081 [−.101, .263]
Beta	**.063**	**.082**	.047	.055	−.021	−.050	.051	.028
Age 41 to 50								
B [95% CI]	.172 [−.040, .385]	.265 [.052, .478]*	.085 [−.152, .322]	.168 [.004, .332]*	−.305 [−.540, −.070]*	−.016 [−.356, .325]	.085 [−.091, .262]	.088 [−.095, .271]
Beta	.050	**.076**	.022	**.062**	**−.080**	−.003	.031	.030
Age 51+								
B [95% CI]	.572 [.358, .787]***	.485 [.269, .700]***	.335 [.096, .574]**	.290 [.124, .456]**	−.057 [−.294, .181]	.934 [.590, 1.278]***	.314 [.136, .493]**	.425 [.240, .611]***
Beta	**.165**	**.139**	**.087**	**.107**	−.015	**.162**	**.113**	**.146**

*Note*. *p < .05, **p < .01, ***p < .001. B = unstandardized regression coefficient, Beta = standardized regression coefficient. Male served as the reference group for gender, White for race/ethnicity, and < 31 years old for age

Results were largely similar after adjusting for an additional set of controls (beyond the “givens” of life), although the magnitude of the associations generally attenuated (see Supplemental Table S2). For example, there was little evidence in support of higher purpose among participants aged 41 to 50 years compared to those aged 31 years or below after controlling for additional sociodemographics, lifestyle, and work-related factors. However, adjustment for non-immutable characteristics means that such attenuation may be due to confounding, mediation, or some combination of both. For this reason, we focus our interpretation on the results from the primary analysis.

## Discussion and Conclusions

Although there is abundant research on the relationship between demographics and specific domains of well-being, such as health [31, 32], this paper is one of the first to examine demographic differences in secure flourishing, or complete well-being. Only two variables predicted this outcome: Asian and the highest age category (51 years and older). However, Hispanics did have a higher level of flourishing when the financial security domain was excluded. We also disaggregated flourishing into six domains: emotional health, physical health, purpose, character strengths, social connectedness, and financial security. Relative to Whites, well-being levels are higher for Asians, Blacks, and Hispanics for most domains, except for financial security, which is lower for Blacks and Hispanics, and physical health, which is higher only for Asians. These results are consistent with the literature on the “paradox” of well-being among groups like Asians, Blacks, and Hispanics that experience high levels of social stressors but also higher levels of well-being [15, 27–29].

It is not uncommon for groups to score highly on some measures of well-being but not as satisfactorily on others. For example, one study found that Blacks had higher levels of psychological and social flourishing than Whites, but also higher levels of mortality [33]. Similarly, our findings disclosed an advantage for Blacks and Hispanics on the psychosocial dimension of well-being, but an advantage for Whites on the material dimension of financial security [6]; see also [34] on materialist vs. postmaterialist concerns]. For our respondents, this distinction helps to account for why Blacks and Hispanics did not exhibit higher secure flourishing. Asians, on the other hand, reported a combination of advantages with regard to both psychosocial and material aspects of well-being, which resulted in a significantly higher score on flourishing *and* secure flourishing.

But complexity emerged even for the high-scoring Asians once we considered gender differences. As a group, Asian females had well-being scores that were always higher than the means for the total sample and the highest overall for three domains as well as the combined measure relative to the other racial/ethnic and gender groups. Asian males, on the other hand, actually scored lower than the total sample on social connections and character strengths, with the latter score being the lowest of all of the racial/ethnic and gender groups. Although this group generally reported greater flourishing on both the psychosocial and material dimensions, as well as on the combined measure, there may be value in further investigation of their lower than average scores on social connectedness and character strengths. Research on Asians has found that social connections are related to other aspects of physical and emotional well-being [35]. Connections and character are likely related to each other as well, in light of the research finding that “strengths of the ‘heart’ (kindness, love) are consistently linked to happiness, whereas strengths of the ‘mind’ (curiosity, open-mindedness) are not” [36].

In other words, the character strengths that foster high quality relationships -- such as love, kindness, and social intelligence -- may be especially relevant over the life course as the luster of social status, intellectual accomplishments, and other strengths of the mind begin to fade. Culture is also an important consideration, as people from interdependent cultural backgrounds tend to value social harmony and emotional support from close others more than those from comparatively individualistic cultures [8, 37]. Our regression results did not find a gender difference in social connectedness, but previous research using different but related survey items indicated that women have higher scores on positive relations with others [22]. Asian females in our sample had the highest score of any sub-group on social connectedness and a character strengths score that was, on average, over a half-point higher than Asian men. Although we are discussing a rather small group, the Asian men in our sample are quite financially secure (they scored the highest of any sub-group), but they also have lower scores on social connectedness and character strengths.

With the exception of physical health, respondents over 50 years of age had higher levels of well-being in all domains and on our two combined measures [38, 39]. This may be related to the robust finding that older adults are more likely enjoy their work, engage with collegial coworkers, and become employed in organizations in which they feel comfortable [40]. In other words, our results may be partly a function of sampling only employed older adults, rather than including older adults who may have exited the job market due to health problems or high dissatisfaction with their work situation. In the general population, happiness appears to be declining for adults over 30 years of age, possibly due to increasing individualism adversely affecting social support [41]. We found a U-shaped relationship for emotional health for those over 30 years old. Relative to our reference category (30 and under), respondents between 31 and 40 years had moderately higher emotional health, then there was a decline to non-significance between 41 and 50, followed by a strong significant effect for 51 and older. Although speculative, this pattern could be due to the aforementioned lack of social support adding to the stresses of parenting teenagers, the onset of a midlife crisis, and/or the “sandwich” (Miller, 1981) experience of having to care for both children and ailing parents, all of which may reach a peak between ages 41 and 50 and then move towards resolution.

This study is not without limitations. First, the sample was drawn from a single organization in a particular sector of the for-profit economy and therefore does not represent the broader population, which would include non-working adults as well as employees who selected a different economic sector (e.g., nonprofit, government, education, finance, hospitality). The oldest age groups, including retirees, are also not represented. Our sample was generally representative of the workforce at the organization under study, but our modest response rate is a further limitation, although it was well-within norms for survey research. Second, it is possible that the findings of this study may be biased due to unmeasured confounding. For immutable “givens” of life including those that we examined, adjusting for non-immutable factors assessed later in life (e.g., educational attainment, income) can be problematic if there is a reasonable likelihood that they might be intervening variables. Hence, we made an a priori decision not to adjust for socioeconomic, lifestyle, or work-related factors reported at the time of data collection (i.e., in adulthood). Nevertheless, it is often standard practice to include such factors and our results were largely, though not entirely, similar after adding these predictors. Furthermore, the analytic rigor of this study was limited by the type and quantity of survey items that were administered. The plausibility of the confounding control assumption might have been strengthened if we were able to retrospectively assess and adjust for potential confounds occurring prior to an individual’s conception [42].

A third limitation is that our findings were derived from a single time point and we do not know whether well-being levels for our respondents remain stable over time. Additionally, our findings are based on self-reports which may be inaccurate or biased by social desirability considerations. Prior research has uncovered that Eastern Asians score higher on the Marlowe-Crowne Social Desirability scale [43], and women generally display greater social desirability traits than men [44], which may partially account for the higher levels of well-being reported, across domains, among Asian Females. Future research can compare reported levels of well-being across a domain to a more objective measure, such as medical claims data for the physical health domain, as an example. We also had relatively small numbers for some of the groups, especially Asian, Black, and Hispanic men, given that over 80% of our sample was female. We were also not able to disaggregate our ethnic data into specific sub-groups (e.g., Chinese, Filipino, Vietnamese). Future studies might also explore the relationship between neighborhood and domains of flourishing, especially in light of the well-established importance of place for domains such as physical health [45]. Finally, it is important to assess the applicability of the domains of well-being across cultures, as the recently launched Global Flourishing Study, led by Gallup, Harvard University, and Baylor University, seeks to do.

Although it is important not to overgeneralize and we caution against using this study’s findings for applied purposes without carefully considering its limitations, we highlight some potential implications for practice. Our findings suggest that relatively brief, multidimensional measures of well-being could provide organizations with an opportunity to develop a more comprehensive understanding of how different groups of employees are doing in multiple key domains of life. Group differences in this study were heterogenous across domains of well-being, which would not have been revealed by more narrow or generalized measures of well-being. To illustrate, we found little evidence to suggest that males and females differed on secure flourishing, but a closer examination of the well-being domains indicated that females tended to score lower on the financial security domain and higher on the character strengths domain. By assessing and monitoring employee well-being more comprehensively, organizations that use surveys or other similar approaches to track employee flourishing might be better positioned to (1) identify domains of well-being that groups of employees might be most likely to benefit from receiving additional resources, (2) make well-informed decisions about the kinds of resources that they should make available to support the well-being of their workforce, and (3) assess the effectiveness of organizational resources in fulfilling objectives related to promoting employee well-being. For example, our findings suggest that, within the organizational context in which this study took place, employees below 31 years of age might be especially likely to benefit from resources aimed at fostering a sense of purpose in life compared to older employees. These kinds of insights are unlikely to be gleaned without using measures of multidimensional well-being, which would also be essential for gauging whether organizational resources dedicated to promoting a specific domain of well-being ultimately translate into improved functioning among the group of employees that the organization envisions would benefit the most from those resources.

As our literature review demonstrated, the relationships among demographics and different domains of well-being vary across studies and over time. There is support for a paradox of well-being among disadvantaged groups, although the findings are sometimes uneven. In order to design the most effective interventions to enhance well-being for specific groups, a more consistent body of research findings would be helpful. We have contributed to this project by examining the relationship between six important domains of complete well-being (secure flourishing) and the demographic “givens” of life. Our primary analytic strategy allowed us to avoid concerns about causal ordering in cross-sectional studies by excluding predictors that might be affected by our outcomes and we found that the distinction between psychosocial and material forms of well-being, also labeled materialist and postmaterialist [34], was helpful for understanding the experiences of different racial/ethnic groups. We hope that future research will incorporate these six domains, as well as others such as spiritual and communal well-being [4]. This would provide a more comprehensive understanding of the well-being advantages, and challenges, associated with different demographic groups.

## Supplementary Information

**Additional file 1.**

## Data Availability

Although we are not able to unilaterally release the data at this time, in collaboration with the organization that collected the data for this study we will submit the data set to a public depository at the conclusion of the 5-year funding period, which ends November 14, 2022. Until then, the data are available for scholarly research upon request from the first author, Dr. Matthew T. Lee (matthew_lee@fas.harvard.edu).
