# Supplementary material for: Demographic Predictors of Complete Well-Being
Source: BMC Public Health. 2022 Sep 6;22:1687. doi: 10.1186/s12889-022-13769-7 (PMC9446856; doi:10.1186/s12889-022-13769-7)
Supplement: Supplementary file 1 — Additional file 1. [file 12889_2022_13769_MOESM1_ESM.docx]

**SUPPLEMENTAL MATERIAL**

“Demographic Predictors of Complete Well-Being”

*BMC Public Health*

Matthew T. Lee, Ph.D. (corresponding author: matthew_lee@fas.harvard.edu), Eileen McNeely, Dorota Weziak-Bialowolska, Karen A. Ryan, Kay D. Mooney, Richard G. Cowden, Tyler J. VanderWeele

| Supplemental Table S1  *Missing Data for Primary Study Variables* | | |
| --- | --- | --- |
| Variable | Missing | |
|  | *n* | % |
| Gender | 0 | 0 |
| Age | 0 | 0 |
| Race/ethnicity | 0 | 0 |
| Emotional Health | 90 | 3.81 |
| Purpose | 87 | 3.68 |
| Social Connectedness | 103 | 4.36 |
| Character Strengths | 87 | 3.68 |
| Physical Health | 119 | 5.04 |
| Financial Well-being | 86 | 3.64 |
| Flourish Index | 280 | 11.85 |
| Secure Flourish Index | 303 | 12.82 |

| Supplemental Table S2  *Regression Results for Demographic Predictors of Well-Being Domains After Adjusting for Additional Set of Control Variables* | | | | | | | | |
| --- | --- | --- | --- | --- | --- | --- | --- | --- |
| Predictor | Criterion Variable | | | | | | | |
|  | Emotional Health | Purpose | Social Connectedness | Character Strengths | Physical Health | Financial Security | Flourish Index | Secure Flourish Index |
| Female |  |  |  |  |  |  |  |  |
| B [95% CI] | .146 [-.019, .310] | -.088 [-.249, .073] | -.176 [-.361, .008] | **-.143 [-.276, -.011]*** | .023 [-.168, .213] | **.402 [.133, .671]**** | -.046 [-.181, .088] | .038 [-.100, .176] |
| Beta | .035 | -.021 | -.038 | **-.045** | .005 | **.058** | -.014 | .011 |
| Asian |  |  |  |  |  |  |  |  |
| B [95% CI] | **.290 [.009, .570]*** | .070 [-.204, .344] | .064 [-.249, .378] | **.241 [.018, .464]*** | .202 [-.121, .525] | **.756 [.298, 1.213]**** | .161 [-.069, .392] | **.297 [.058, .536]*** |
| Beta | **.041** | .010 | .008 | **.044** | .026 | **.064** | .028 | **.050** |
| Black |  |  |  |  |  |  |  |  |
| B [95% CI] | **.321 [.122, .521]**** | **.286 [.090, .483]**** | .081 [-.141, .304] | **.344 [.183, .504]***** | .072 [-.160, .303] | **-.509 [-.835, -.183]**** | **.213 [.047, .379]*** | .086 [-.084, .257] |
| Beta | **.069** | **.061** | .016 | **.094** | .014 | **-.065** | **.056** | .022 |
| Hispanic |  |  |  |  |  |  |  |  |
| B [95% CI] | .236 [-.004, .477] | **.257 [.021, .493]*** | **.311 [.039, .584]*** | **.390 [.197, .583]***** | .061 [-.218, .340] | -.240 [-.633, .153] | **.267 [.069, .464]**** | .174 [-.029, .377] |
| Beta | .039 | **.043** | **.046** | **.084** | .009 | -.024 | **.055** | .035 |
| Age 31 to 40 |  |  |  |  |  |  |  |  |
| B [95% CI] | **.266 [.047, .485]*** | **.236 [.020, .451]*** | .243 [-.001, .488] | .134 [-.042, .310] | -.112 [-.364, .141] | -.204 [-.561, .153] | .163 [-.015, .341] | .109 [-.074, .292] |
| Beta | **.077** | **.067** | .063 | .050 | -.029 | -.035 | .058 | .038 |
| Age 41 to 50 |  |  |  |  |  |  |  |  |
| B [95% CI] | .189 [-.040, .418] | .165 [-.060, .390] | .104 [-.152, .359] | .122 [-.061, .305] | **-.383 [-.646, -.119]**** | -.078 [-.451, .295] | .051 [-.135, .238] | .035 [-.157, .228] |
| Beta | .054 | .047 | .027 | .045 | **-.100** | -.013 | .018 | .012 |
| Age 51+ |  |  |  |  |  |  |  |  |
| B [95% CI] | **.437 [.198, .675]***** | **.256 [.022, .490]*** | .139 [-.128, .406] | .132 [-.059, .324] | -.234 [-.510, .041] | **.442 [.053, .832]*** | .140 [-.055, .335] | .187 [-.014, .387] |
| Beta | **.126** | **.073** | .036 | .049 | -.061 | **.076** | .050 | .064 |
| *Note*. **p* < .05, ***p* < .01, ****p* < .001. B = unstandardized regression coefficient, Beta = standardized regression coefficient. Male served as the reference group for gender, White for race/ethnicity, and < 31 years old for age. Each analysis controls for sociodemographics (education [high school (ref), some college, associate degree, bachelor’s degree, graduate degree], marital status [single, not married (ref), married, divorced, widowed, separated, non-married partner], having children below 18 at home [no (ref), yes], home ownership [no (ref), yes]), lifestyle (religious service attendance [daily (ref), not daily but more than once a week, once a week, 1-3 times a month, once every few months, once a year, never]), and work characteristics (job satisfaction [0-10], work-family conflict [0-10], job insecurity [0-10], number of days/week working from home [none (ref), 1, 2, 3, 4, 5]). | | | | | | | | |
